# Ring trial 2016 for Bluetongue virus detection by real‐time RT‐PCR in France

**DOI:** 10.1002/vms3.63

**Published:** 2017-04-16

**Authors:** Corinne Sailleau, Cyril Viarouge, Emmanuel Breard, Damien Vitour, Stephan Zientara

**Affiliations:** ^1^ ANSES/INRA/ENVA‐UPEC UMR 1161 Virologie 14 rue Pierre et Marie CURIE‐94700 Maisons Alfort France

**Keywords:** Bluetongue, diagnosis, real‐time RT‐PCR, ring trial

## Abstract

Since the unexpected emergence of BTV‐8 in Northern Europe and the incursion of BTV‐8 and 1 in France in 2006–2007, molecular diagnosis has considerably evolved. Several real‐time RT‐PCR (rtRT‐PCR) methods have been developed and published, and are currently being used in many countries across Europe for BTV detection and typing. In France, the national reference laboratory (NRL) for orbiviruses develops and validates ‘ready‐to‐use’ kits with private companies for viral RNA detection. The regional laboratories network that was set up to deal with a heavy demand for analyses has used these available kits. From 2007, ring tests were organized to monitor the performance of the French laboratories. This study presents the results of 63 regional laboratories in the ring trial organized in 2016. Blood samples were sent to the laboratories. Participants were asked to use the rtRT‐PCR methods in place in their laboratory, for detection of all BTV serotypes and specifically BTV‐8. The French regional laboratories are able to detect and genotype BTV in affected animals. Despite the use of several methods (i.e. RNA extraction and different commercial rtRT‐PCRs), the network is homogeneous. The ring trial demonstrated that the French regional veterinary laboratories have reliable and robust BTV diagnostic tools for BTV genome detection.

## Introduction

Bluetongue virus (BTV) is the causative agent of Bluetongue (BT), an insect‐transmitted disease of domestic and wild ruminants (Verwoerd & Erasmus 2004; Maclachlan *et al*. 2009). Bluetongue virus is the prototype member of the *Orbivirus* genus in the *Reoviridae* family (Mertens *et al*. 2005) with 27 serotypes currently described (Zientara *et al*. 2014) and at least three putative new serotypes (Maan *et al*. 2015; Sun *et al*. 2016). In 2006, BTV serotype 8 (BTV‐8) emerged unexpectedly in Northern Europe throughout a region including Belgium, France, Germany, Luxembourg and the Netherlands. In the following year, it spread rapidly throughout the rest of Europe. During the same period, BTV serotype 1 was also identified in Southern Europe (Portugal, Spain and South West France) (Wilson & Mellor 2009). Following several vaccination campaigns, many Northern European countries recovered their BTV‐free status, including France (in December 2012). However, BTV‐8 unexpectedly re‐emerged in France in September 2015 (Sailleau *et al*. 2015). These European incursions of BTV have caused considerable economic losses at multiple levels: firstly, direct losses in sheep and cattle from mortality, morbidity, reduced production, abortions and fetal malformations in calves (only for BTV‐8) (Vercauteren *et al*. 2008; Zanella *et al*. 2012) and secondly, indirect losses resulting from ensuing bans on trade in ruminants between areas where the BTV is or not circulating (Council Directive 2000/75/EC and Commission Regulation EC No 1266/2007 3). In view of the significance of the disease, all affected countries have established control and eradication measures requiring the availability of detection and prevention tools, such as efficient inactivated vaccines.

During this major BTV‐8 epizootic (2006–2010), real‐time RT‐PCR (rtRT‐PCR) was the main diagnostic tool used in Europe for the detection of BTV in infected ruminants, to enable the authorities to implement sanitary measures and control the disease. These molecular tools are used in the event of clinical suspicions, animal movements or prevalence studies involving mainly domestic but also wild ruminant species.

Several rtRT‐PCR assays have been developed and published and are currently being used in many countries across Europe (Jimenez‐Clavero *et al*. 2006; Orru *et al*. 2006; Shaw *et al*. 2007; Toussaint *et al*. 2007a; Hofmann *et al*. 2008). Since 2007, the French National Reference Laboratory or NRL (ANSES) has collaborated with four private companies (Thermo Fisher Scientific, Adiagène, IDvet Genetics and Biosellal) to develop and validate RNA extraction protocols and ‘ready‐to‐use’ rtRT‐PCR kits for the detection of the BTV genome from ruminant EDTA blood samples (Zientara *et al*. 2015). All these kits are able to detect the genome of all serotypes including the three novel serotypes (BTV‐25 to BTV‐27). In order to cover the demand for diagnoses, a network of 63 regional laboratories was mobilized. As BTV‐8 and BTV‐1 were circulating simultaneously in France in 2007–2008, specific rRT‐PCRs for both serotypes were developed by the same companies and validated by the French NRL to track the spread of these viruses. These kits and methods were then decentralized in the regional labs. Every 2 years, the French NRL is asked by the DGAL (Directorate General for Food) to organize a ring trial to measure the performance of the network laboratories for BTV group‐specific detection and serotype‐specific detection. This paper reports the results of the ring trial organized in 2016. As only BTV‐8 was circulating in France, the laboratories were asked to perform rtRT‐PCRs for the detection of all serotypes and specifically for the detection of BTV‐8.

## Materials and methods

### Participating laboratories

Sixty‐three laboratories from all over France participated in this ring trial. A code number was assigned to each laboratory to ensure a blind analysis of the proficiency test.

### Sample preparation

An EDTA blood sample from non‐infected cattle was spiked (or not) with supernatants from cell culture infected with BTV‐8 and BTV‐1. Four different master tubes (with four different viral loads) of BTV‐8 and one of BTV‐1 were prepared. Ultimately, the panel contained 10 samples numbered 1 to 10, two negative samples, one of BTV‐1, and 7 of BTV‐8 (one in triplicate, one in duplicate and two individuals) (Table 1). Each sample was divided into 450 *μ*l aliquots to produce 80 batches of 10 samples. The batches were stored at −80°C until control and submission. Each tube was randomly coded with a unique number.

**Table 1:** Panel of blood samples prepared for the ring trial

Samples	Nature	Pan‐BTV rtRT‐PCR mean CT value (Standard deviation)	BTV‐8 rtRT‐PCR mean CT value (Standard deviation)
1/2	Non spiked blood	No CT	No CT
3/4/5	BTV‐8 spiked blood	30.4 (0.7)	31.0 (1)
6	BTV‐8 spiked blood	28.7 (0.5)	28.6 (0.9)
7	BTV‐8 spiked blood	32.0 (0.7)	32.4 (2.3)
8/9	BTV‐8 spiked blood	32.7 (0.8)	32.9 (1.8)
10	BTV‐1 spiked blood	29.4 (0.7)	No CT

rtRT‐PCR results (mean CT values and standard deviation) for each sample, all rtRT‐PCR kits taken together

### Control of samples before submission

Homogeneity of samples was evaluated from five tubes of each sample following the procedure NF ISO 13528. Total RNA was extracted from 100 *μ*L of blood using the robotic workstation ‘QIAcube’ and the QIAamp Viral RNA kit (Qiagen; reference: 52906) according to the manufacturer's instructions. After RNA denaturation (3 min at 95°C), rtRT‐PCR was performed in duplicate using the commercial real‐time RT‐PCR kit Adiavet™ BTV REAL TIME (reference ADI‐352, Bio‐X Diagnostics) according to the manufacturer's instructions. The coefficient of variation (CV ‐ the ratio of the standard deviation to the mean) was estimated for each sample. Stability was assessed from three tubes of each sample as soon as the last laboratory sent in its results (after 8 weeks at −80°C and 1 week at +4°C).

A batch of samples was tested with every commercial kit used by the French laboratory network (Table 2). Total RNA was extracted from samples using a robotic method (Kingfisher) with the MagVet Universal Isolation kit, and RNAs were tested with each commercial rtRT‐PCR kit. The CT value cut‐off is 40 for all kits.

**Table 2:** Commercial rtRT‐PCR kits available for the ring trial

rtRT‐PCR assays	Commercial kits
Duplex Pan BTV rtRT‐PCR (detection all serotypes + endogenous gene)	LSI VetMax Bluetongue Virus NS3
	ADIAVET BTV REAL TIME (ADI352)
	ID Gene^®^ BlueTongue duplex
	Bio‐T kit^®^ BTV all genotypes
Duplex BTV‐8 rtRT‐PCR (detection of BTV‐8 + endogenous gene)	LSI VetMax BTV 8 typing‐IAH
	ADIAVET BTV TYPE 8 REAL TIME (ADI381)
Triplex Pan BTV rtRT‐PCR + BTV‐8 rtRT‐PCR (detection all serotypes + BTV‐8 + endogenous gene)	ADIAVET BTV ALL + TYPE 8 REAL TIME (ADI401)

### Ring trial recommendations

An authorised carrier was mandated for the shipping of blood samples (at +4°C) to the 63 laboratories. Participants were asked to use the rtRT‐PCR methods in place in their laboratory, for detection of all BTV serotypes and specifically BTV‐8. They were also asked to provide details about the RNA extraction, the rtRT‐PCR kits and the thermocycler used. Results were due 7 days after receipt of the samples. For each coded sample, the laboratory had to indicate on the results form, the CT values and the results ‘detected/non detected’ for both pan‐BTV and BTV‐8 rtRT‐PCRs.

## Results

### Sample preparation and control

#### Homogeneity and stability

Five batches of 10 samples were tested in duplicate using the NRL's routine method. The coefficient of variation results obtained for each sample was inferior to 3%.

For the stability assessment, three batches were tested in duplicate, after 8 weeks at −80°C and 1 week at +4°C. The difference in mean CT values between the first test of homogeneity and these tests was <1 for all samples. The stability was then considered as satisfactory.

CT values with all commercial rtRT‐PCR kits.

Blood samples were extracted with a robotic method (Kingfisher) and RNAs were tested with each commercial kit. All BTV‐8 spiked blood were positive using the Pan‐BTV rtRT‐PCR and BTV‐8 rtRT‐PCR kits. The BTV‐1 spiked blood was positive only with the Pan‐BTV rtRT‐PCR. The non‐spiked blood was negative with all kits. The mean CT values and the standard deviation for each sample were calculated and are presented in Table 1.

#### Ring trial results

**Figure 1:**
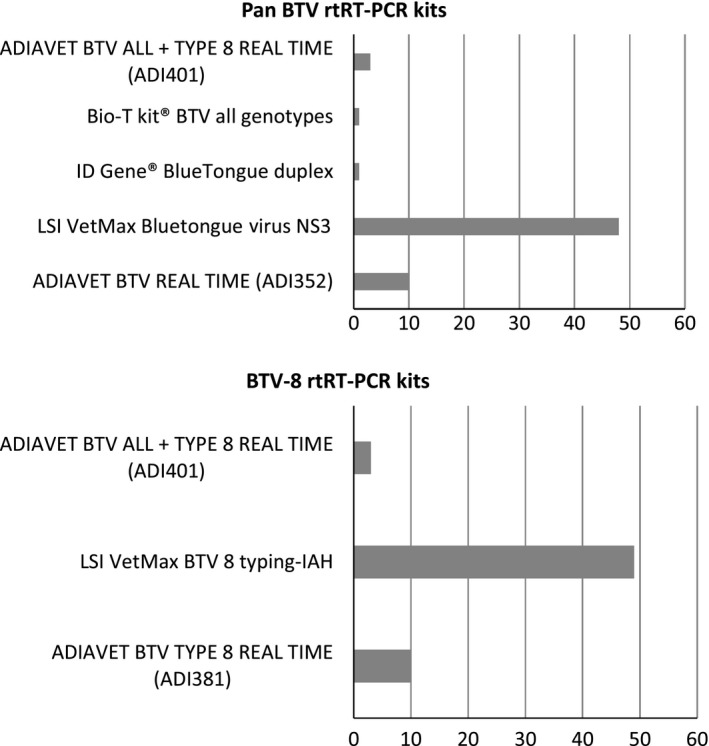
rtRT‐PCR kits used by participants.

All laboratories returned the results form within the timeframe (1 week). For the RNA extraction, the majority of labs (40/63) used a robotic method based on magnetic beads (e.g. KingFisher/MagMax) with different commercial kits (e.g. MagVet Universal Isolation kit). In 23 of the 63 participating laboratories, the nucleic acids were extracted using a method based on silica membrane technology: 20 used a manual method and three an automated method (Qiacube or Qiaextractor) with commercial kits (e.g. QIAamp VIRAL RNA/Nucleospin96Virus Core kits). Participants were asked to use the commercial rtRT‐PCR kit they had in their laboratory and that was commonly used for BTV detection (Fig. 1).

For all laboratories, negative samples (Samples 1 and 2) and all positive samples (Samples 3–10) were tested correctly, respectively ‘non‐detected’ and ‘detected’, with the pan‐BTV rtRT‐PCR kits. With the exception of one participant that failed to detect a BTV‐8 sample (Sample 9), the BTV‐8 typing by BTV‐8 rtRT‐PCR was correct for all laboratories. The sample 9, together with sample 8, were spiked with the smallest viral load.

**Figure 2:**
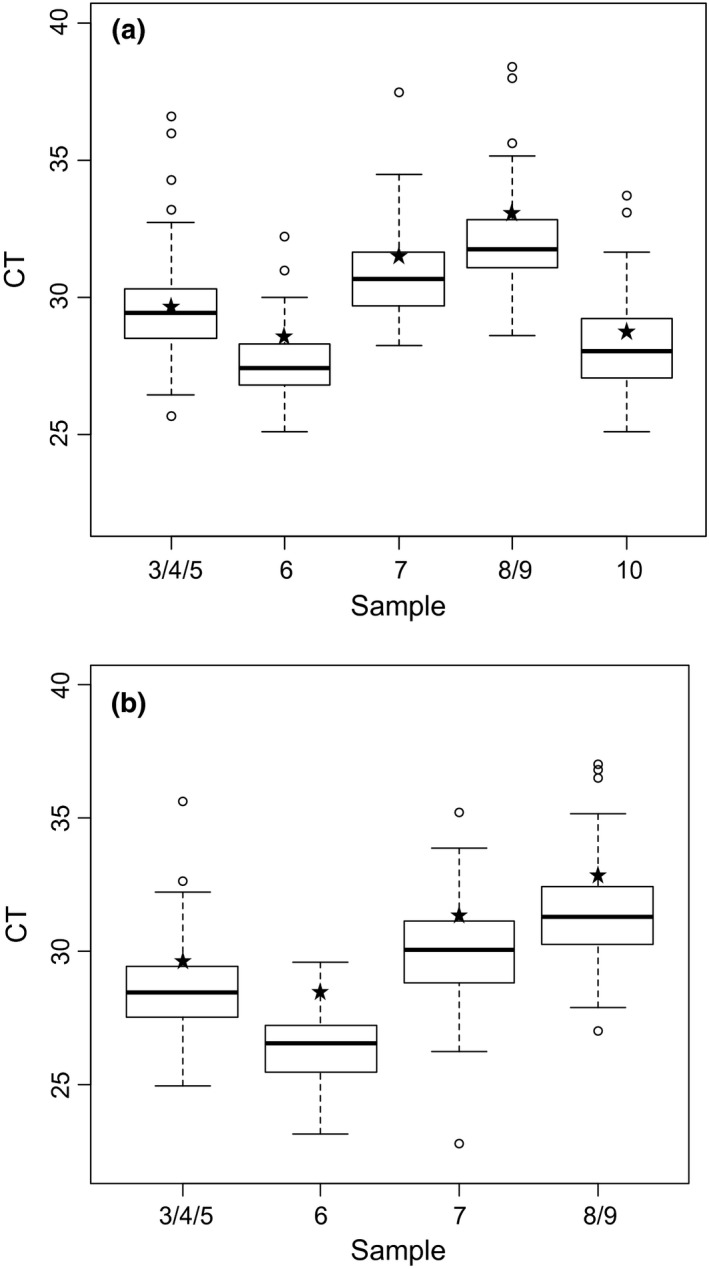
Box plot of real‐time RT‐PCR results of the positive samples generated with all the methods used by the 63 laboratories for BTV detection (a) and for BTV‐8 detection (b). White circles represent the extreme CT values obtained by some laboratories. Asterisks represent the NRL results.

**Figure 3:**
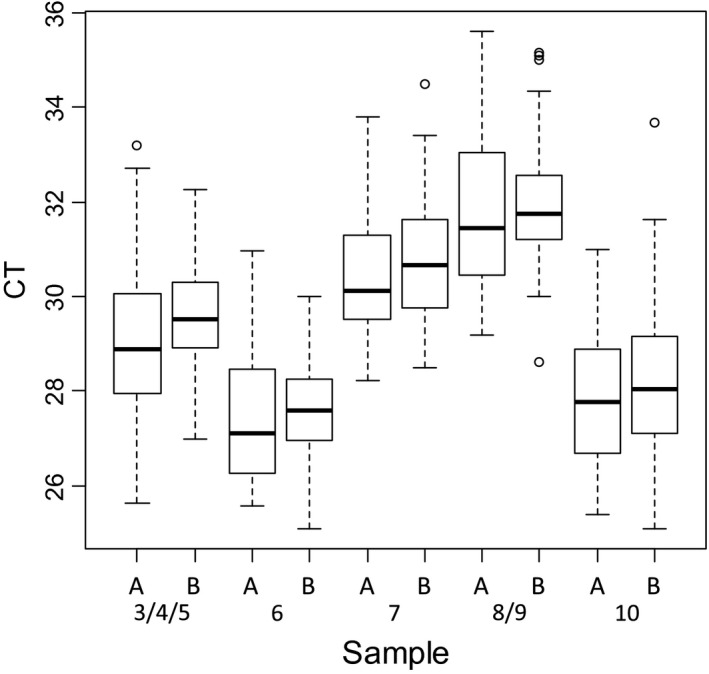
Box plot of pan‐BTV real‐time RT‐PCR results of the positive samples depending on the extraction methods used (A: silica membrane, B: magnetic beads).

The CT values were analyzed for each sample whatever the method used (Fig. 2) and depending on the extraction method (silica membrane ‐manual or automatic‐, or magnetic beads) (Fig. 3).

For both pan‐BTV and BTV‐8 detection and for each sample, the variability of the interquartile range is homogeneous (approximatively 2 CT). The extreme CT values do not exceed 4 CT to the median (Fig. 2). A slight deviation of CT values was observed for each sample extracted with magnetic beads (a mean CT value of +1) (Fig. 3).

The mean, standard deviation and coefficient of variation were also calculated for each positive sample and are shown in Table 3. The coefficients of variation ranged between 4.6 and 6.1% and 4.9 and 6.8% respectively for pan‐BTV detection and BTV‐8 detection.

**Table 3:** rtRT‐PCR results for pan‐BTV detection and BTV‐8 detection

** **	Pan BTV rt‐RT‐PCR kits				BTV‐8 rtRT‐PCR kits			
Samples	CT (mean)	SD	CV	N of values	CT (mean)	SD	CV	N of values
3/4/5	29.6	1.5	5.1	189	28.5	1.5	5.2	186
6	27.6	1.3	4.6	63	26.4	1.3	4.9	62
7	30.8	1.6	5.2	63	30,0	2.0	6.8	62
8/9	32.0	1.5	4.8	126	31.4	1.8	5.6	123
10	28.2	1.7	6.1	63				

Mean CT values, standard deviation and coefficient of variation are given for each sample (all method taken together).

### Discussion

Before 2006, the molecular diagnosis of BTV was performed in France by conventional RT‐PCR. During the BTV epizooty that occurred in Corsica between 2000 and 2004 (Gerbier *et al*. 2008), genome detection was carried out by conventional RT‐PCR, targeting segment 7 (Wade‐Evans *et al*. 1990; Zientara *et al*. 2002). Only the NRL (ANSES Maisons‐Alfort) was in charge of the diagnosis. In 2006, following the major epizootic in Northern Europe (OIE 2006, Toussaint *et al*. 2006, 2007b), our laboratory and the CODA‐CERVA (Belgium NRL) joined forces to develop and validate two rtRT‐PCR methods targeting segments 1 and 5 (Toussaint *et al*. 2007a). In 2007, to deal with the heavy demand for analyses (clinical suspicion, animal movements), a network of 67 regional laboratories was formed and was able to test hundreds of thousands of blood samples per day in good conditions. The network first used the rtRT‐PCR (segment 1) method developed in‐house, then in 2008, this technique was replaced by ‘ready‐to‐use’ rtRT‐PCR kits developed and validated in collaboration with two private companies (Zientara *et al*. 2015). All developed kits were either in duplex or triplex format targeting both BTV gene(s) and an endogenous gene, able to amplify a portion of ruminant GAPDH or beta‐actin RNA. The simultaneous amplification of an endogenous control gene as an internal control attests to the quality of the analyzed sample and the absence of PCR inhibitors.

Between 2007 and 2009 a ring trial was organized each year, but since 2009 it has been performed only once every two years. In 2016, 63 laboratories participated in the ring trial. A total of seven commercially available rtRT‐PCR kits were used by the participating laboratories in combination with different methods of RNA extraction (manual or automatic based on silica membrane, and automatic based on magnetic beads) (Fig. 1). For all laboratories, results of the pan‐BTV rtRT‐PCR kits were correct for each sample. With the exception of one participant (which failed to detect the BTV‐8 Sample 9), the typing by BTV‐8 rtRT‐PCR of BTV‐8 genome was correct for all laboratories. This lab was contacted to elucidate the reason of the lack of sensitivity, and a problem with the temperature of the heating block used for RNA denaturation was identified. A new sample panel was provided and all BTV‐infected blood samples were then correctly identified.

Although the laboratories were assessed on their capacity to detect the presence or absence of BTV genome (qualitative results), they were also asked to provide CT values for each sample. The qualitative results showed that the laboratory network performed excellently for the detection and typing of BTV‐8. No problem of false positive results related to contamination (which is a known risk in molecular diagnostics) was observed. The CT values were analyzed to estimate the dispersion of CT values across the network (using different combinations of RNA extraction processes and rtRT‐PCR kits) (Fig. 2). The coefficients of variation do not exceed 6.8% for pan‐BTV detection and BTV‐8 detection (Table 3). Laboratories that showed high CT values (more than 4 CT from the median) were also contacted to help them improve the detectability of BTV (CT values of these labs appear as white circles in the box plot in Figure 2).

Some variability in CT values was expected and can be explained by differences in extraction RNA quality and yield of purified RNAs, a suboptimal denaturation of RNA, the PCR instruments and commercial rtRT‐PCR kits used by the laboratories. The CT values were also analyzed for each sample depending on the RNA extraction technology used (manual or automated based on silica membrane, or magnetic beads with robots).The slight deviation of CT values observed for each sample extracted with magnetic beads (a mean CT value of +1) (Fig. 3) is not necessarily significant. Indeed, the methods (RNA denaturation/rtRT‐PCR kits) used by the laboratories for amplification of RNA extracted were different and it is possible that other parameters (than RNA extraction) may have an impact on the CT values.

Taken together, the ring trial results showed that the French regional laboratories have a good ability to detect BTV in affected animals despite the use of different methods (RNA extraction and commercial rtRT‐PCR kits). The development and validation of ‘ready‐to‐use’ kits meant that the network of laboratories could be mobilized speedily to assist in the management of animal movements. Indeed, in 2007, the spread of BTV‐8 to the centre of France led to a ban on the trade from France to the North of Italy: every autumn more than 500,000 cattle are transported from France to Italy for fattening (Zientara *et al*. 2015). The use of the network allowed the departure of thousands of animals after they were tested by rtRT‐PCR. After the re‐emergence of BTV‐8 in France in September 2015 (Sailleau *et al*. 2015), all laboratories were asked to participate in an epidemiological survey in order to determine the prevalence of the infection. Moreover, hundreds of thousands of RT‐PCR tests are now performed in France to diagnose BTV infection and control animal movements in France (movements from restricted areas to BTV‐free areas) or for export. This requires having a homogeneous network, which is why a ring test is organised regularly. In order to obtain more information on the appropriateness of the current PCR kits to certify ruminants as free of BTV, it would be of interest to include in the next ring trial (in 2018), samples of a wider range of viral RNA load (at the lower limit of the detection spectrum).

In conclusion, the ring trial revealed that the French regional veterinary laboratories were capable of reliable and robust BTV diagnostics for BTV genome detection.

## Source of Funding

This work was funded by the Laboratory's own resources.

## Conflict of Interest

The authors have no conflict of interest to declare

## Ethics Statement

The authors confirm that the ethical policies of the journal, as noted on the journal's author guidelines page, have been adhered to. No ethical approval was required as no animal was used in this study.’

## Contributions

Coordination of the ring trial: CS; Preparation/sending of samples: CS/CV/EB; Analysis of the results: CS/CV; Article writing: CS/EB/SZ/DV.
